# Impact of *myo*‐inositol trispyrophosphate (ITPP) on tumour oxygenation and response to irradiation in rodent tumour models

**DOI:** 10.1111/jcmm.14092

**Published:** 2018-12-21

**Authors:** Ly‐Binh‐An Tran, Thanh‐Trang Cao‐Pham, Bénédicte F. Jordan, Sofie Deschoemaeker, Arne Heyerick, Bernard Gallez

**Affiliations:** ^1^ Biomedical Magnetic Resonance Group, Louvain Drug Research Institute Université catholique de Louvain Brussels Belgium; ^2^ Normoxys Inc. Boston Massachusetts

**Keywords:** EPR oximetry, hypoxia, ITPP, oxygen consumption, radiotherapy

## Abstract

Tumour hypoxia is a well‐established factor of resistance in radiation therapy (RT). *Myo*‐inositol trispyrophosphate (ITPP) is an allosteric effector that reduces the oxygen‐binding affinity of haemoglobin and facilitates the release of oxygen by red blood cells. We investigated herein the oxygenation effect of ITPP in six tumour models and its radiosensitizing effect in two of these models. The evolution of tumour pO_2_ upon ITPP administration was monitored on six models using 1.2 GHz Electron Paramagnetic Resonance (EPR) oximetry. The effect of ITPP on tumour perfusion was assessed by Hoechst staining and the oxygen consumption rate (OCR) in vitro was measured using 9.5 GHz EPR. The therapeutic effect of ITPP with and without RT was evaluated on rhabdomyosarcoma and 9L‐glioma rat models. ITPP enhanced tumour oxygenation in six models. The administration of 2 g/kg ITPP once daily for 2 days led to a tumour reoxygenation for at least 4 days. ITPP reduced the OCR in six cell lines but had no effect on tumour perfusion when tested on 9L‐gliomas. ITPP plus RT did not improve the outcome in rhabdomyosarcomas. In 9L‐gliomas, some of tumours receiving the combined treatment were cured while other tumours did not benefit from the treatment. ITPP increased oxygenation in six tumour models. A decrease in OCR could contribute to the decrease in tumour hypoxia. The association of RT with ITPP was beneficial for a few 9L‐gliomas but was absent in the rhabdomyosarcomas.

## 1 INTRODUCTION

Hypoxia has long been recognized as a critical factor in tumour growth and response to therapy.^1^, ^2^, ^3^ Tissue oxygenation is determined by the balance between oxygen delivery to tissue and oxygen consumption by tissue. This balance is well‐maintained in normal tissues, whereas solid tumours are unable to maintain the balance as a result of the aberrant structure and function of tumour vessels^4^, ^5^ and the intense metabolism rate in tumour cells.^6^ In some cases, cancer cells can use adaptive responses to escape oxygen deficiency and stimulate the selection for clonogenic cells with increased hypoxia tolerance. The expansion of these cell clones can, in turn, aggravate tumour hypoxia, thereby establishing a vicious circle of increasing hypoxia and subsequent malignant progression. Translated in the clinic, this vicious circle leads to more local recurrences, loco‐regional spread, distant metastases and greater resistance to cancer therapies.^7^ Particularly, tumour oxygen status has been shown to be important for outcome following radiation; cancer patients with hypoxic tumours have been reported to be at higher risk for radiotherapy failure.^8^, ^9^

Considering the compelling link between tumour hypoxia and treatment outcome, efforts have been made to develop effective hypoxia‐targeted therapies.^8^, ^9^, ^10^, ^11^, ^12^, ^13^, ^14^ Among these, using hypoxia modifier that can improve tumour oxygenation during irradiation represents one of the most attractive strategies. Recently, ITPP (*myo*‐inositol trispyrophosphate), has been suggested to exert such activity. This compound has intrinsic anti‐cancer properties as it has demonstrated therapeutic efficacy in various animal models when used alone or combined with chemotherapies.^15^, ^16^, ^17^, ^18^, ^19^ ITPP is an allosteric effector that reduces the oxygen‐binding affinity of haemoglobin and may thus facilitate the oxygen release by red blood cells.^20^, ^21^ This effect as well as a potential vascular normalization through the down‐regulation of HIFs/VEGF may alleviate tumour hypoxia.^15^, ^17^, ^18^ Starting from these previous studies, the present study was designed to answer the following questions: (i) is the expected increase in tumour oxygenation upon ITPP treatment a common feature among several cancer models?; (ii) what is the time window of increase in oxygenation induced by ITPP?; (iii) could changes in perfusion and oxygen consumption rate (OCR) potentially contribute to the effect of ITPP?; (iv) could ITPP improve tumour outcome when combined with radiotherapy?

## 2 MATERIALS AND METHODS

### 2.1 Tumour models and ITPP treatment

Four mouse tumour models (mouse fibrosarcoma FSaII implanted in C3H mice, mouse mammary tumour NT2 in FVb/Nrj mice, human breast cancer MDA‐MB‐231 in NMRI nude mice and human cervix squamous cell carcinoma SiHa in NMRI nude mice) and two rat tumour models (rat 9L‐glioma in Fischer F344 rats and rat rhabdomyosarcoma in WAG/Rij rats) were used. The origin of cell lines and animals were reported elsewhere.^22^, ^23^, ^24^, ^25^ Tumours were inoculated subcutaneously in the thigh of animals according to the protocols described previously.^22^, ^23^, ^24^, ^25^ Experiments were performed when tumours reached a diameter of 6‐8 mm (in mouse models) or 14‐16 mm (in rat models). ITPP (kindly provided by Normoxys Inc) was injected intraperitoneally. Doses ranged from 0.5 to 4.0 g/kg. ITPP solution was prepared by dissolving the compound in injectable water to the target concentration and adjusted to pH = 7 by using a small volume of 0.1 mol/L NaOH. The experimental design included various dosage of ITPP and various treatment schedules to explore which regimen could offer the best oxygenation effect.

### 2.2 Tumour oxygenation

An L‐bandEPR spectrometer (Magnettech, Berlin, Germany) operating at 1.2 GHz was used to evaluate the dynamic change in tumour oxygenation upon ITPP administration. Charcoal suspension (CX0670‐1; EM Science, Gibbstown, NJ, USA; 100 mg/mL), used as the oxygen sensor, was introduced intratumourally (about 60 µL for a mouse tumour and 200 µL for a rat tumour) 1 day before the experiment. The charcoal is dispersed over the whole tumour. During EPR recording, animals were anaesthetized with 2% isoflurane in air and their body temperature was maintained at 37 ± 1°C using a circulating warm water system. This anaesthesia regimen was previously demonstrated not to disturb the haemodynamics in rodents.^26^ The pO_2_ measurements were carried out 15 minutes after the induction of the anaesthesia. The linewidth of the first‐derivative EPR spectrum that was the average of five 1‐minute scan accumulations was then converted to pO_2_ using a calibration curve.^27^

### 2.3 Tumour perfusion

Hoechst 33342 (Sigma) staining was used to assess 9L‐glioma perfusion 1 day after completion of the treatment (2 g/kg ITPP once daily for 2 days). Rats were killed exactly 2 minutes after intravenous injection of Hoechst solution (15 mg/kg in saline). Tumour fragments were rapidly excised, embedded in optimal cutting temperature compound and frozen in liquid nitrogen‐cooled isopentane. Frozen sections of 5 μm thickness were photographed using a Zeiss Mirax fluorescence microscope and images were analysed using Frida software. The percentage of tumour perfusion was calculated as the ratio of Hoechst‐positive area to the total area of tumour sections (no necrosis was histologically detected at this stage of tumour development).^28^

### 2.4 Tumour cell oxygen consumption rate

The impact of ITPP on OCR was assessed on six cell lines (FSaII, SiHa, MDA‐MB‐231, NT2, 9L‐glioma and rhabdomyosarcoma) using a Bruker EMX X‐band EPR spectrometer operating at 9.5 GHz and ^15^N‐PDT (^15^N 4‐oxo‐2,2,6,6‐tetramethylpiperidine‐d_16_‐^15^N‐1‐oxyl, CDN isotopes; Pointe‐Claire, Quebec, Canada) as the oxygen sensor. Cells were incubated with 10 mmol/L ITPP for a period of 2 or 6 hours. LY294002 (Invitrogen), a PI3K inhibitor, was also included in the study at a concentration of 20 µmol/L to compare its effect with ITPP. After harvest, cells were resuspended in culture medium at a concentration of 10^7 ^cells/mL. About 100 µL of cell suspension was mixed with 100 µL of 20% dextran to avoid agglomeration and then sealed in a glass capillary tube in the presence of 0.2 mmol/L ^15^N‐PDT. Cells were maintained at 37°C during the acquisition of the spectra. EPR linewidth was measured every minute and reported on a calibration curve to obtain the oxygen concentration. OCR was determined by the slope of the decrease in oxygen concentration in the closed capillary tube over time.^29^, ^30^

### 2.5 Irradiation

The regimen of 2 g/kg ITPP once daily for 2 days was used. Irradiation was performed 2 hours after the second administration of ITPP (time at which tumour oxygenation was shown to be highest). Two rat models (9L‐glioma and rhabdomyosarcoma) were employed; rats were randomly divided into four groups: vehicle, ITPP, RT + vehicle and RT + ITPP. Single dose of irradiation was delivered by a ^137^Cs irradiator IBL‐637 (Oris, France), 20 Gy for rhabdomyosarcoma and 30 Gy for 9L‐glioma. Animals anaesthetized with isoflurane (2% in air) were placed on a plexiglass and protected from the beam through a lead block of 3 cm thickness while the tumours were exposed through a hole 25 mm in diameter. The animals were turned midway through the exposure time to enhance the uniformity of dose distribution. Irradiation doses were selected on the basis of their respective radiation sensitivity to ensure a significant growth delay in the irradiation group compared to the untreated one. Treatment effect was analysed based on tumour growth delay assay. Tumours were measured on the starting day of treatment to determine the initial size and then at least twice a week until the end‐point (time at which a tumour doubled its initial diameter). Clonogenic assays were also performed to evaluate the radiosensitization effect of ITPP on rhadomyosarcoma and 9L‐glioma cell lines (Supplementary Materials and Methods).

### 2.6 Statistical analysis

All results were expressed as mean ± SEM. Differences between groups were analysed using *t* test or Mann‐Whitney test when data were not normally distributed. Log‐rank test was used to compare Kaplan‐Meier curves. *P* < 0.05 was considered statistically significant for all tests.

## 3 RESULTS

### 3.1 Impact of ITPP on tumour oxygenation and treatment schedule optimization

**Figure 1:**
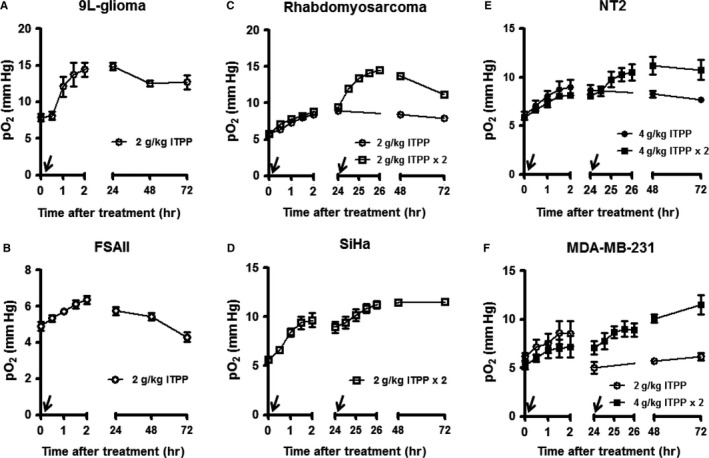
Impact of ITPP administration on tumour oxygenation as measured by EPR oximetry in six tumour models. The arrow indicates the injection of ITPP. (n = 3‐6/group)

A first screening of pO_2_ evolution upon ITPP administration was conducted on various tumour models to assess the effect of this compound on tumour oxygenation. The first test with 9L‐glioma and FSaII showed a significant increase in tumour pO_2_, quickly within 2 hours after the injection of a single dose of 2 g/kg ITPP (*P* = 0.0002 and *P* = 0.0014, respectively) (Figure 1A,B). The oxygenation increase observed in 9L‐glioma (83.6%) was much larger than in FSaII (29.6%). This effect was maintained for 1 day before slowly returning to baseline levels. In the next model, rhabdomyosarcoma, another administration of 2 g/kg ITPP was added on the following day that further enhanced the effect (Figure 1C). The second increase in pO_2_ after the second dose of ITPP was also found in SiHa model (Figure 1D). Given the moderate oxygenation effect on the first four models, we applied a doubled dose of ITPP (4 g/kg) to mice bearing NT2 tumours. However, only a limited increase in pO_2_ (from 6 to maximum 12 mm Hg) was obtained (Figure 1E). Similarly, we observed the accumulative effect in MDA‐MB‐231 model upon the second injection; however, 2 hours after the first injection, the group of 4 g/kg had no advantage over that of 2 g/kg (Figure 1F).

**Figure 2:**
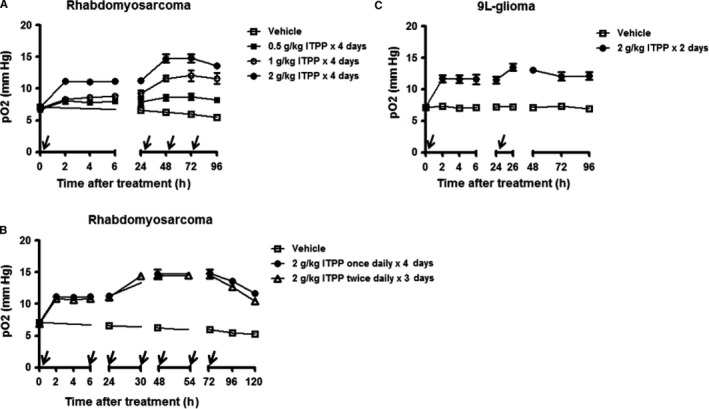
Optimization of ITPP treatment schedule. (A) Various doses of ITPP from 0.5 to 2 g/kg were given daily to rats bearing rhabdomyoscoma for 4 consecutive days. (B) The effect of once‐daily and twice‐daily treatment of ITPP on rhadomyosarcoma was compared. For the former regimen, ITPP was given at 0, 24, 48 and 72 h; for the later regimen, ITPP was given at 0, 6, 24, 30, 48 and 54 h. (C) Verifying the optimal schedule (2 g/kg ITPP once daily for 2 days) on 9L‐glioma. The arrow indicates the injection of ITPP. (n = 4‐6/group). Each point is the mean of measurements done in different animals

Rhabdomyosarcoma was then used to optimize the treatment schedule of ITPP. To investigate if lower doses of ITPP could induce a similar increase in oxygenation, various doses of ITPP ranging from 0.5 to 2 g/kg were injected once a day within 4 consecutive days. The results showed an obvious relationship between the dose and the response (Figure 2A). Dose of 2 g/kg offered the optimal effect; however, the third and fourth doses did not induce any additional effect. The effect of once‐daily and twice‐daily treatment was then compared. In the twice‐daily regimen, the second dose of the day was given at 6 hours after the first one, the treatment was lasting for 3 days. So overall, the animals of this group received six doses of 2 g/kg in 3 days; whereas, the animals of the once‐daily treatment group received four doses of 2 g/kg in 4 days. No difference between two groups was observed (Figure 2B). Hence, 2 g/kg once daily for 2 days was considered as the optimal regimen of ITPP treatment. This optimal schedule was finally verified on 9L‐glioma. The result showed the most elevated tumour oxygenation at 2 hours after the second injection of ITPP as expected (Figure 2C).

### 3.2 Contributing factors to increase in oxygenation: Effect of ITPP on tumour perfusion and OCR

**Figure 3:**
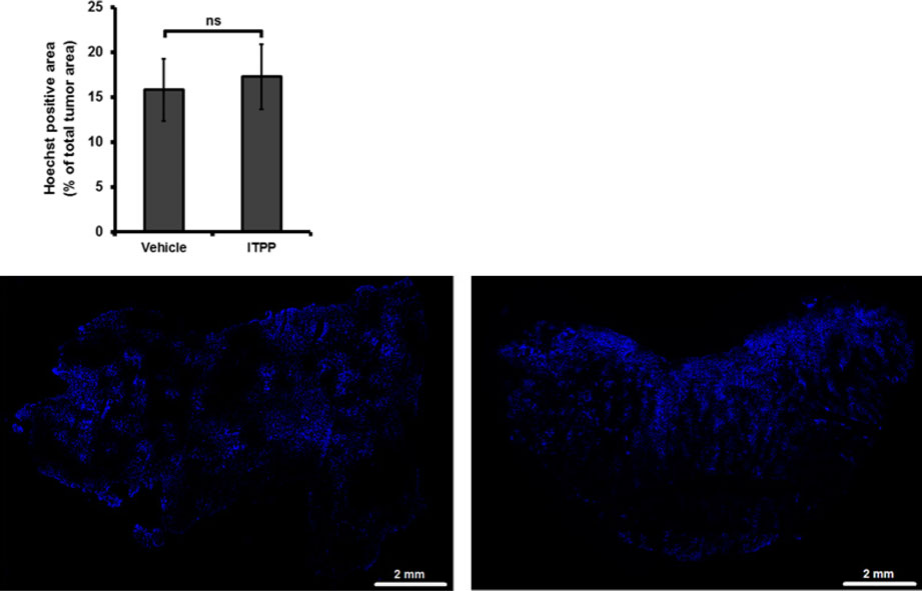
Impact of ITPP treatment (2 g/kg once daily for 2 days) on 9L‐glioma perfusion as assessed by Hoechst 33342 staining. Top panel: No significant difference in Hoechst‐perfused area was found between the groups with and without ITPP treatment. “ns” = not significant (n = 4‐5/group). Bottom panel: Representative Hoechst fluorescence images of an untreated (left) and a treated tumour (right)

**Figure 4:**
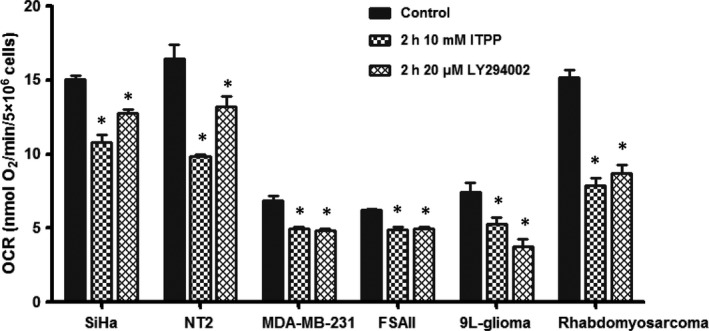
Impact of ITPP on oxygen consumption rate in vitro. *P < 0.05 when comparing the treated group with the control group (n = 3‐8/group)

Tumour perfusion 1 day following the optimal treatment (2 g/kg of ITPP once daily for 2 days) was assessed on 9L‐glioma (Figure 3). Areas of perfusion, corresponding to areas stained by Hoechst 33342, were not significantly different between the groups with and without ITPP treatment. The impact of this compound on OCR of cancer cells was then studied. As shown in Figure 4, exposure to 10 mmol/L ITPP in 2 hours significantly inhibited OCR in all six cell lines. No further benefit was found when expanding the incubation time to 6 hours (data not shown). Interestingly, the PI3K inhibitor LY294002 also induced a comparable effect at the similar timing.

### 3.3 Radiosensitization effect of ITPP

**Figure 5:**
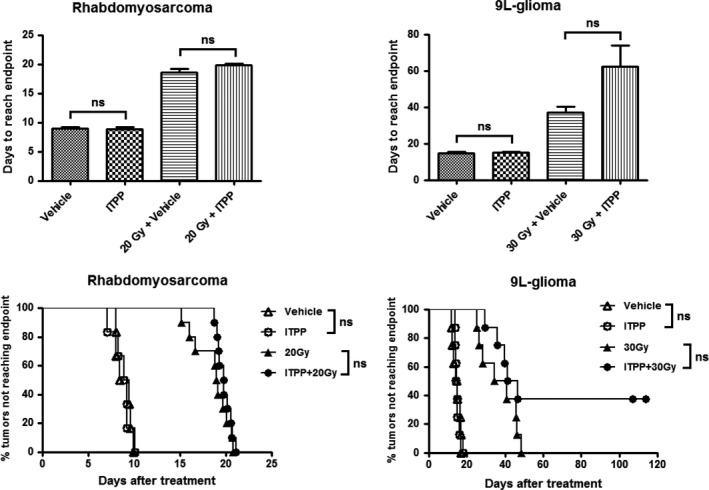
Effect of ITPP treatment (2 g/kg once daily for 2 days) on tumour growth of rhabdomyosarcoma and 9L‐glioma. Top panel: Bar graphs showing the time for tumours to reach the end‐point. Bottom panel: Kaplan‐Meier curves showing the percentage of tumours that did not reach the end‐point. For the cured tumours, the ending day of experiment was taken as the value of tumour growth time. “ns” = not significant. (n = 6‐10/group)

To investigate the radiosensitization effect of ITPP, we combined the optimal treatment schedule of ITPP (2 g/kg once daily for 2 days) with irradiation of 20 and 30 Gy on rhabdomyosarcoma and 9L‐glioma, respectively. The results of tumour growth delay assay are presented on Figure 5. In both models, ITPP monotherapy did not affect tumour growth, as the times to reach the end‐point were 8.80 ± 0.42 days (n = 6) and 8.90 ± 0.35 days (n = 6) for the treated and untreated rhadomyosarcomas, 15.00 ± 0.53 days (n = 8) and 14.61 ± 0.71 days (n = 8) for the treated and untreated 9L‐gliomas respectively. When combined with irradiation, ITPP did not improve the outcome in rhabdomyosarcomas: 19.83 ± 0.24 days for the RT + ITPP group (n = 10) vs 18.61 ± 0.63 days for the RT + vehicle group (n = 10). In the case of 9L‐glioma, the variation between individual responses was high within the group irradiated plus ITPP. Two different categories of response were observed in this group: one did not benefit from ITPP treatment and the other has been completely cured with no tumour recurrence by the end of the experiment (n = 3). This is highlighted in the Kaplan‐Meier curves (Figure 5 bottom panel). As a consequence of this heterogeneity of response, the tumour growth time of the RT + ITPP group was not statistically different compared to the RT + vehicle group (62.26 ± 11.69 days, n = 8 vs 36.99 ± 3.41 days, n = 8). Additionally, data from clonogenic assays showed that ITPP treatment did not induce any significant intrinsic radiosensitization (Figure S1).

## 4 DISCUSSION

*Myo*‐inositol trispyrophosphate has demonstrated therapeutic efficacy in a wide range of animal models^15^, ^16^, ^17^, ^18^, ^19^ and shown safety in a Phase I human clinical trial (http://normoxys.com/clinical-trial-results/). Using the OxyLite system, Raykov et al found that a single ITPP injection induced an increase in partial pressure of oxygen for almost 1 week in pancreatic tumour xenografts.^16^ In another study, Kieda et al observed a similar effect in B16 melanoma model and 4T1 mammary tumour model.^15^ Based on the previous results, we decided to explore the potential benefit when combining ITPP with radiotherapy. First, we assessed the effect of ITPP on tumour oxygenation in a larger panel of tumour models and tried to define the optimal dose/schedule regimen leading to a maximal increase in tumour oxygenation. For that purpose, we used EPR oximetry,^31^, ^32^ a non‐invasive and highly sensitive technique that can provide the quantitative absolute values of pO_2_ in vivo and is able to repeat the measurements at the same site over long periods of time. Our results obtained by EPR indicated that ITPP treatment quickly increased tumour oxygen levels in the six tested models and reached the maximum effect at 2 hours post‐administration. Such elevated pO_2 _was sustained for at least 1 day and then gradually decreased (Figure 1). Interestingly, a cumulative effect was found when the second ITPP administration was given with a 24‐hours interval, but additional administration did not lead to further increase in tumour pO_2_ (Figure 2). It should be noted that the effect of ITPP was rather moderate compared to those previously reported.^15^, ^16^ This may be because of the difference in response of different tumour models but also because of the methods used to measure oxygen tension as the previous studies were carried out using OxyLite. OxyLite provides dynamic oxygen measurement at a single point inside tissue, whereas EPR reports pO_2 _on a larger volume as the oxygen sensor is dispersed over the whole tumour (Figure S2).

Regarding the mechanism, ITPP is an allosteric effector that reduces the oxygen‐binding affinity of haemoglobin and thus facilitates the oxygen release by red blood cells.^20^, ^21^ In addition, long‐term ITPP administration has been shown to lead to vascular normalization through the down‐regulation of HIFs/VEGF with a consequent increase in the oxygen supply to tumours.^15^, ^17^, ^18^ To determine possible contributing factors to the increase in oxygenation, we assessed the effect of ITPP on tumour perfusion and on cancer cell OCR. Considering the quick increase in tumour pO_2_ within 2 hours of treatment, vessel normalization would not be expected as a mode of action for the effect observed herein; however, an increase in blood volume could not be excluded. Our data from Hoechst staining did not show any change in tumour perfusion in 9L glioma model. We next evaluated if OCR could be modulated by ITPP. We observed that ITPP significantly inhibited OCR in all six cell lines. Interestingly, ITPP has been shown to inhibit PI3K,^15^ and the inhibition of PI3K pathway has been proposed to reduce the cell OCR.^33^, ^34^ We suggest that the effect of ITTP on OCR could be comparable to other inhibitors of PI3K such as LY294002. We observed that both compounds induced similar effects on the OCR of six cell lines at the same timing. Our results demonstrated that ITPP was an inhibitor of tumour cell respiration and identified the inhibition of OCR as another contributing mechanism to the ITPP‐induced increase in oxygenation. At this stage, the mechanism supporting the effect of ITTP on OCR remains unknown and the hypothesis of an effect mediated by an inhibition of the PI3K remains to be validated in future studies. Of note, it has been suggested theoretically^35^ and experimentally^36^ that in order to alleviate tumour hypoxia, decreasing oxygen consumption should be more efficient than increasing oxygen delivery. Interestingly, in the study of Kelly et al, blockade of PI3K pathway was found to reduce OCR and to increase tumour oxygenation despite no change in overall perfusion.^33^ This result was quite in line with what we observed in the present study. A possible limitation of our approach is that OCR was measured in vitro. Although the reduced OCR of cancer cells exposed to ITPP was correlated to the increase in tumour oxygenation in terms of timing, in vitro results may not translate perfectly to in vivo system.^37^ It will thus be very interesting in the future to assess the impact of ITPP on OCR in vivo. For this purpose, ^17^O magnetic resonance spectroscopy (^17^O‐MRS) would be the most appropriate choice to observe the modulation of tumour OCR by ITPP.^25^ Multiple mechanisms may play a determining role at an early stage, and then the others may become predominant at a later stage. In this case, the non‐invasive magnetic resonance imaging (MRI) based methods, such as R2*‐MRI to detect deoxyhaemoglobin content^38^, ^39^ and Dynamic Contrast‐Enhanced MRI to characterize vascular network,^40^, ^41^ would be helpful to further explore the mode of action of ITPP over the course of treatment.

Finally, the anti‐tumour properties of ITPP were investigated on rhabdomyosarcoma and 9L‐glioma whose radiosensitivity has been shown to be correlated with oxygenation level.^42^, ^43^, ^44^ When used as a single therapy, ITPP showed no influence on tumour growth in these two models. This result was not in accordance with those observed previously on melanoma, hepatoma and pancreatic cancer where tumour growth was dramatically delayed thanks to long‐term treatment with weekly ITPP.^15^, ^16^, ^17^ However, differences in treatment schedules make the comparison of the data in the present paper with those published earlier very tenuous. We then combined ITPP treatment with irradiation to explore if the increase in oxygenation induced by ITPP could lead to a radiosensitization. We did not observe any benefit from the association ITPP + RT in rhabdomyosarcoma. In 9L‐glioma model, a trend towards an increase in the response was found when combining ITPP with irradiation; however, the response was highly heterogeneous. Indeed, some 9L‐gliomas were completely cured, whereas the other tumours did not respond any better. In comparison with our previous study,^45^ carbogen breathing was able to radiosensitize a majority of 9L‐gliomas and the degree of response was significantly correlated with oxygen level. Such correlation could not be found herein, suggesting that ITPP‐induced increase in oxygenation may not be the determinant factor affecting tumour outcome. In fact, many factors and mechanisms may contribute to the ultimate efficacy of radiotherapy. Besides oxygenation status, ability of repairing DNA damage and cancer cell repopulation following irradiation are also believed to be an important cause of treatment failure.^46^, ^47^, ^48^ If ITPP actually works via a molecular pathway related to PI3K signaling, then the radiosensitizing effect of ITPP would be more complicated than just a simple decrease in hypoxia. PI3K pathway is a key regulator of various cellular functions from cell proliferation to cell survival and is implicated in all major mechanisms of radioresistance.^49^, ^50^ Several studies have indicated the strong involvement of PI3K pathway in repairing the radiation‐induced DNA double‐strand breaks through DNA‐dependent protein kinase.^51^, ^52^, ^53^ In non‐small cell lung cancer, treatment using PI3K/Akt inhibitors could change the apoptotic potential of cancer cells and counteract cell survival, resulting in a better radiosensitivity.^54^, ^55^ However, this effect was found only in tumours and cell lines with high level of PI3K/Akt activation. Similarly, targeting PI3K could only promote radiation‐induced apoptosis in breast cancer cell lines in which this pathway is overstimulated.^56^ Regarding the therapeutic efficacy of ITPP, it should be emphasized that our study is not the first that reported the disappointing result of ITPP. The recent work combining ITPP with radiotherapy on mice bearing GL261^57^ and another one using long‐term ITPP treatment on rats bearing RG2 glioblastoma^58^ have both pointed out the failure of this compound. The fact that not all tumours could benefit from ITPP treatment suggests that the anti‐cancer properties of ITPP may be based on a specific signaling which is not ubiquitously expressed. To verify this hypothesis, another study will be needed to identify the main pathway and key elements that are critical for driving the action of ITPP. To mimic more closely clinical irradiation protocols, it would be interesting to use fractionated irradiation instead of a single irradiation dose as used in the present study.

In summary, our data consistently demonstrated the increased tumour oxygenation in six animal models upon ITPP administration. We also showed in a proof of concept experiment that the enhancement in oxygen level likely resulted from a decrease in oxygen consumption rather than an increase in oxygen perfusion at least at the early stage. The increase in tumour oxygenation induced by ITPP only partly radiosensitized one of the two investigated models. Taking our finding together with the previous reports from the literature, ITPP possesses to some extent potential characteristics that can be beneficial to cancer treatment. However, to take full advantage of its capacity and to move further into clinical trials, a complete picture on the mode of action of this compound is mandatory. Future studies should focus on the underlying mechanism of ITPP and on how the oxygenation effect would be involved in the ultimate therapeutic efficacy of ITPP.

## CONFLICT OF INTEREST

The authors confirm that there are no conflicts of interest.
