# Supplementary material for: Impact of myo‐inositol trispyrophosphate (ITPP) on tumour oxygenation and response to irradiation in rodent tumour models
Source: J Cell Mol Med. 2018 Dec 21;23(3):1908–16. doi: 10.1111/jcmm.14092 (PMC6378184; doi:10.1111/jcmm.14092)
Supplement: Supplementary file 1 [file JCMM-23-1908-s001.doc]

**Supplementary Materials and Methods:**

**Clonogenic assay**

Clonogenic survival was performed to evaluate the radiosensitization effect of ITPP on rhabdomyosarcoma and 9L-glioma cell lines. Cells were irradiated using a ^137^Cs irradiator IBL-637 (Oris, France) at a dose rate of 1.1 Gy/min for a total dose of 2, 4, 6 and 8 Gy. Immediately after irradiation, cells were detached using trypsin-EDTA, plated at different densities into 6-well plates and incubated at 37°C to allow the formation of colonies. After 10 days of incubation, cells were fixed and stained with crystal violet for 30 min. Colonies of at least 50 cells were counted. The surviving fraction was calculated as the ratio of the plating efficiency of the irradiated cells to that of non-irradiated cells. For the ITPP-treated group, cells were incubated with 10 mM ITPP for 2 hours before and during the irradiation.

**Supplementary Figures**

| 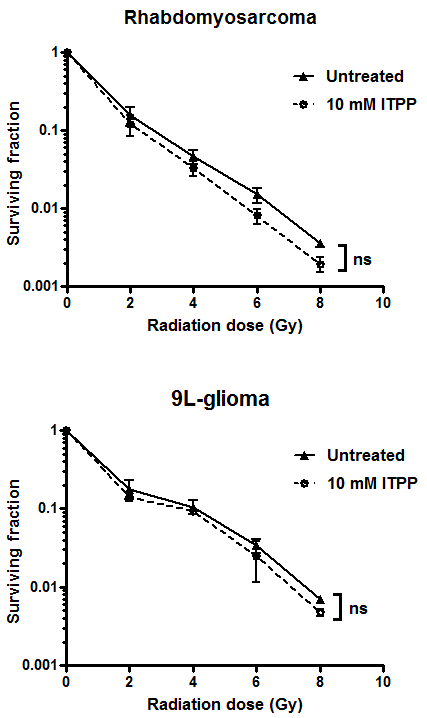 |
| --- |
| **Fig. 1S.** Surviving fraction of rhabdomyosarcoma and 9L-glioma cells after irradiation with and without ITPP treatment. “ns” = not significant. |
| **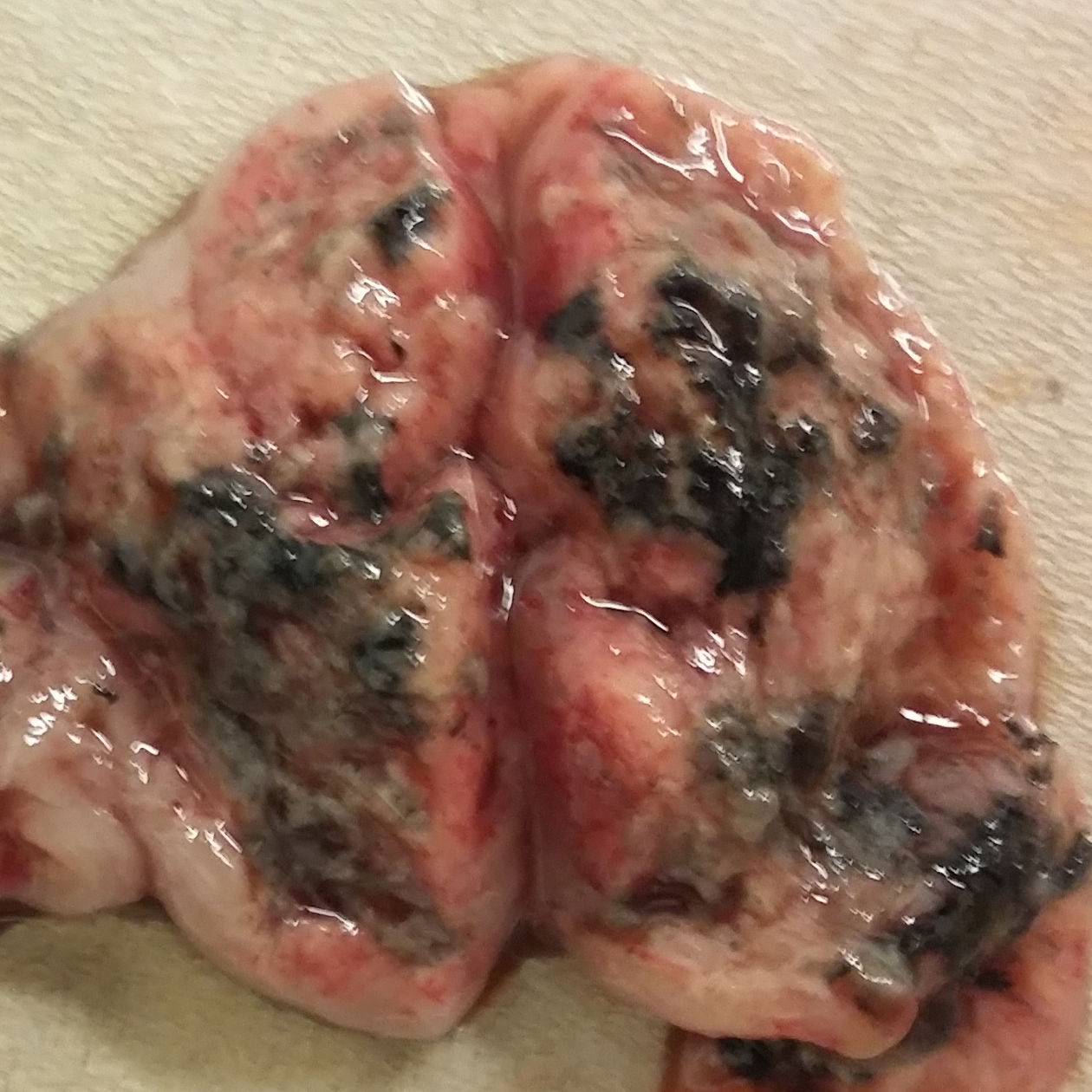** |
| **Fig. 2S.** Distribution of charcoal within a rat tumor. |
